# Avian responses to an extreme ice storm are determined by a combination of functional traits, behavioural adaptations and habitat modifications

**DOI:** 10.1038/srep22344

**Published:** 2016-03-01

**Authors:** Qiang Zhang, Yongmi Hong, Fasheng Zou, Min Zhang, Tien Ming Lee, Xiangjin Song, Jiteng Rao

**Affiliations:** 1 Guangdong Public Laboratory of Wild Animal Conservation and Utilization, Guangdong Entomological Institute/South China Institute of Endangered Animals, Guangzhou 510260, China; 2 Guangdong Key Laboratory of Integrated Pest Management in Agriculture, Guangdong Entomological Institute/South China Institute of Endangered Animals, Guangzhou 510260, China; 3 Woodrow Wilson School of International and Public Affairs, Princeton University, Princeton NJ 08544, USA; 4 Guangdong Chebaling National Reserve, Shixing 512500, China

## Abstract

The extent to which species’ traits, behavior and habitat synergistically determine their response to extreme weather events (EWE) remains poorly understood. By quantifying bird and vegetation assemblages before and after the 2008 ice storm in China, combined with interspecific interactions and foraging behaviours, we disentangled whether storm influences avian reassembly *directly* via functional traits (i.e. behavioral adaptations), or *indirectly* via habitat variations. We found that overall species richness decreased, with 20 species detected exclusively before the storm, and eight species detected exclusively after. These shifts in bird relative abundance were linked to habitat preferences, dietary guild and flocking behaviours. For instance, forest specialists at higher trophic levels (e.g. understory-insectivores, woodpeckers and kingfishers) were especially vulnerable, whereas open-habitat generalists (e.g. bulbuls) were set to benefit from potential habitat homogenization. Alongside population fluctuations, we found that community reassembly can be rapidly adjusted via foraging plasticity (i.e. increased flocking propensity and reduced perching height). And changes in preferred habitat corresponded to a variation in bird assemblages and traits, as represented by intact canopy cover and high density of large trees. Accurate predictions of community responses to EWE are crucial to understanding ecosystem disturbances, thus linking species-oriented traits to a coherent analytical framework.

Climate variations drive ecological and evolutionary responses in most taxonomic or functional groups, however predicting climate-induced changes in community assembly, behavioral adaptation and ecosystem functioning are core challenges for ecology^1^,^2^,^3^,^4^. Extreme weather events (EWE) including ice storms, wildfires, flooding, hurricanes, and drought represent extreme disturbances to ecosystems because they alter habitat structure and resource availability^5^. Beyond direct effects on populations, EWE may affect community dynamics through (i) the specific characteristics/functional traits of individual species^6^,^7^, (ii) interactions among species^8^,^9^, and (iii) synergies with habitat modifications^10^,^11^. Most studies have concentrated on the effects of EWE on the populations of individual species. Yet the potential effects of EWE on community assemblages as well as changes in the biotic interactions and behavioral adaptations of species, though important, are seldom considered. Compounded with logistical difficulties, the unpredictability of EWE and the lack of pre-event data or replications make studying the community effects of EWE challenging.

Ice storms are highly destructive disturbances that have the potential to influence floral and faunal communities. In early 2008, an extreme ice storm event occurred across a large geographical band in southeastern China, causing massive mechanical damage to native broad-leaved forests. China’s State Forestry Administration (SFA) estimated that the storm damaged 20.86 million hectares-one-tenth of China’s forests and plantations. SFA pegs the losses at $8 billion^12^. Freezing rain resulted in heavy ice accumulation on the branches and trunks of forest trees^13^. In the worst affected forests, most trees were uprooted or had their trunks snapped, while the few standing trees were stripped of most if not all of their branches^14^,^15^,^16^. The heavy storm also resulted in mortality among birds and animals, many of them frugivorous^17^. Rapid biodiversity surveys reported that the population densities of butterflies (e.g. Pieridae and Papilionidae species)^18^,^19^, birds (e.g. Silver Pheasants *Lophura nycthemera*, Golden Pheasant *Chrysolophus pictus*)^20^,^21^,^22^, and arthropods (e.g. Hymenoptera, Symphyla species)^23^ significantly declined in severely damaged areas. Given that the forests affected by the 2008 storm were subject to fixed monthly monitoring programs (pre-/post- EWE), this event presented us with a unique opportunity to study the ecological effects of EWE-induced damages on community assembly, ecosystem functioning and behavioral adaptations of bird species.

Determining the effects of EWE, both direct and indirect, on local community assemblages represents a significant challenge. Specifically, (i) Biodiversity responses to EWE include both direct effects on biological parameters of a species (e.g. mortality, reproductive rates, and life history traits), and indirect modifications of relationships between species and their habitats^1^,^24^,^25^. (ii) Functional groups or guilds differ systematically in their sensitivity to climate variations^6^,^26^,^27^, and a sudden EWE shock may considerably modify the functional traits of the community^28^,^29^, likely resulting in the biotic homogenization of ecological communities^30^,^31^,^32^. Explicitly testing the local dynamics of habitat specialists versus generalists in response to EWE is therefore essential. (iii) EWE may influence species assemblages with both positive and negative effects as different species will respond to disturbance in different ways^33^,^34^,^35^,^36^. In addition, species exhibit “mixed” responses to EWE, that is, individual species may exhibit some combination of positive, neutral, and negative responses when tallied across studies^29^. (iv) Regional climatic variation creates selective pressure on the evolution of locally adapted physiologies, and behavioral adaptations over the long-term (e.g. foraging strategies and breeding systems)^9^,^37^, while the short-term effects of EWE might be difficult to discern when individuals and populations within communities display some degree of adaptive abilities and/or phenotypic plasticity^38^. Unfortunately, these inconsistencies across studies are difficult to reconcile (e.g. a lack of pre-EWE data and little treatment of replication/controls).

Assessing the relationship between EWE and the spatial and temporal distributions of both bird and vegetation assemblages with EWE could provide critical information on community responses. Such a study would provide a stronger base of inferring cause-and-effect between an EWE and community changes. We adopted a before–after/control–impact (BACI)^39^ approach in which both bird and vegetation characteristics were simultaneously measured at the same set of points at independent sites both before (from Jan to Dec 2007) and after (from March 2008 to March 2009) the 2008 storm event in Southern China. This made it possible to compare changes in avian/vegetation structure, species interaction and foraging behavior before and after the 2008 storm in the same area. Specifically, we addressed the following hypotheses: (i) Species-specific characteristics (e.g. taxon, trophic level, functional guild) may influence the ecological responses to storm-induced disturbances, which may tend to result in homogenization of ecological communities; (ii) After the storm, community reassembly is accompanied by a rapid readjustment of bird biotic interactions and behavioral adaptations (i.e. interspecific flocking behaviour, preferred perching height); (iii) Drastic changes in vegetation structure following the storm could explain the trend in forest bird assemblages, thus the functional traits of the bird community could be used as indicators to monitor and evaluate habitat restoration.

## Results

### Vegetation structure

After the storm, canopy trees, including Box-leaved Syzygium (*Syzygium buxifolium*), Sweet Gum (*Liquidambar formosana*), Red Oatchestnut (*Castanopsis hystrix*), Fabers Chestnut (*C. fabric*), Chinese Red Pine (*Pinus massoniana*), Itea (*Itea chinensis*) and Chinese Spicebush (*Lindera communis*) were severely damaged thus altering the composition and structure of the plant community. The vegetation destruction varied among quadrats depending on species, diameter at breast height (DBH), tree height, and vegetation layers. Comparison of pre- and post-storm vegetation metrics revealed a significant decrease in both species richness and abundance of large arbors above 12 m in height (Wilcoxon Signed Ranks Test for richness, *Z* = −2.388, *df* = 7, *P* = 0.017; for abundance, *Z* = −2.207, *df* = 7, *P* = 0.027), and tree height (*Z* = −3.646, *df* = 7, *P* < 0.001). Meanwhile, variables associated with understory shrub-grass layers (e.g. species richness, relative abundance, height and cover) tended to increase after the storm event, although these differences were not significant (Table 1). In sum, the arborous species that were dominant and made up the dominant layer of the forest canopy were the most affected.

### Patterns of change in bird species and relative abundance of functional-guild

**Figure 1:**
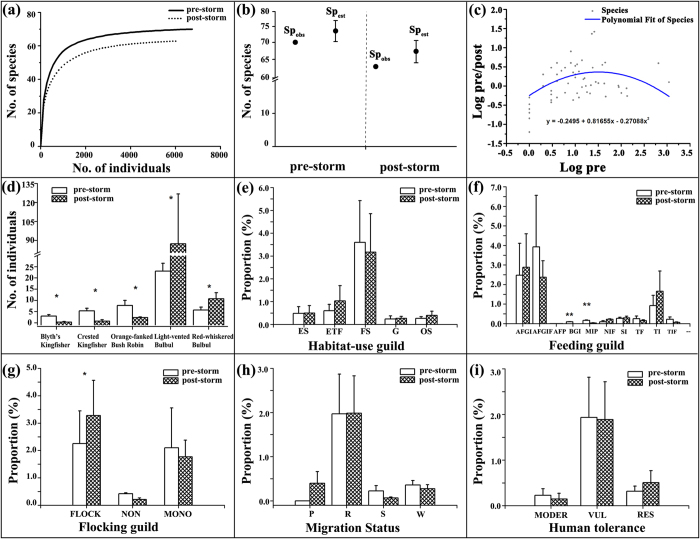
(**a**,**b**) Rarefaction curves and estimates of bird species richness during sampling periods pre- and post- storm, at Chebaling, Guandong province, China; (**c**) Regression plot fitted by Generalized Linear Model with Maximum Likelihood showing the population fluctuation (Log pre-/post-storm) of species; (**d**) Comparison of five bird species that exhibited significant population change after extreme ice storm; (**e**–**i**) Changes in the proportion of relative abundance of bird species grouped by (**e**) habitat preference, (**f**) dietary guild, (**g**) flocking guild, (**h**) migratory status and (**i**) human tolerance. The abbreviation refers to: Habitat preference: “edge species (ES)”, “edge-tolerant forest species (ETF)”, “forest specialist (FS)”, “generalist (G)”, “open-habitat species (OS)”; Dietary guilds: “arborous foliage glean insectivore (AFGI)”, “arborous foliage glean insectivore-frugivore (AFGIF)”, “arboreal frugivore–predator (AFP)”, “bark-gleaning insectivore (BGI)”, “miscellaneous insectivore-piscivore (MIP)”, “nectarivores-insectivore-frugivore (NIF)”, “sallying insectivore (SI)”, “terrestrial frugivore (TF)”, “terrestrial insectivore (TI)”, “terrestrial insectivore-frugivore (TIF)”; Flocking guilds: “mixed-species flock participants (FLOCK)”, “Non-flocking species (NON)” and “monospecific flock participants (MONO)”; Migratory status: “passage migrant (P)”, “permanent resident (R)”, “summer visitor (S)” and “winter visitor (W)”; Human tolerance: “moderate susceptibility (MODER)”, “vulnerable to human disturbance (VUL)” and “resistant to human disturbance (RES)”. Level of significance: *P < 0.05; **P < 0.01.

Sampling saturation was achieved for both pre- and post-storm, as indicated by their rapid approach to asymptote (Fig.1a), meaning we are confident that the vast majority of species present were recorded, and more species were expected and observed pre-storm (Fig. 1b). At ice-affected stands, a total of 70 species were detected prior to the storm and 58 afterward, of which 50 species were present in both periods. And of these, 20 species (mainly forest woodpecker and insectivorous) were exclusively detected before the storm, while eight species (mainly edge and open-habitat species) were observed after the storm only (Table S1). Relative abundance decreased significantly in response to the storm (Paired T-Test; *T* = 2.238, *df* = 77, *P* = 0.033). Regression plots obtained from Generalized Linear Model (GLM) Fit demonstrated that the population of rare species declined more than common species (GLM of Log pre/post-storm population decline: *F* = 8.741, df = 75, *P* = 0.0003; *R*^2^ = 16.7%) (Fig. 1c). Moreover, three of these species [Blyth’s Kingfisher (*Alcedo hercules*), Crested Kingfisher (*Megaceryle lugubris*), and Orange-flanked Bush Robin (*Luscinia cyanura*)] decreased, whereas two species [Light-vented Bulbul (*Pycnonotus sinensis*), and Red-whiskered Bulbul (*P. jocosus*)] increased significantly (Fig. 1d, see Table S1 for statistics).

Sixty-six species that were detected frequently enough to include in a statistical analysis showed differences in guild or trait composition from pre- to post-storm. Habitat preference, dietary guild and flocking behaviour underlay bird relative abundance dynamics in response to the storm, whilst migration status and human tolerance were less important (Fig. 1e–i). For habitat preference, only forest specialists declined more than expected by chance alone (*T* = 1.437, *df* = 22, *P* = 0.165), which probably allow for the increase of edge-tolerant and open-habitat species sharing the same ecological niche (Fig. 1e). Diet was strongly associated with extirpated and declining species, with the sharpest declines seen in “bark-gleaning insectivores” (*T* = 8.000, *df* = 4, *P* = 0.001) and “miscellaneous insectivore-piscivores” (*T* = 4.151, *df* = 6, *P* = 0.006) (Fig. 1f). Among the flocking guilds, it should be noted that the propensity of species to flock in mixed-species groups increased significantly after the storm (*T* = 2.736, *df* = 43, *P* = 0.036) (Fig. 1g). In summary, forest-dwelling specialists and species at higher trophic levels were more likely to be lost from bird communities than generalist species or those at lower trophic levels.

### Shifts in bird perching height

**Figure 2:**
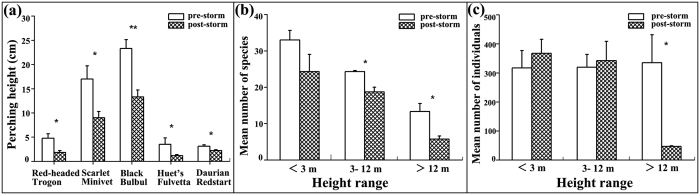
(**a**) Comparison of five bird species that exhibited significant decline in perching height after the storm; (**b**,**c**) Changes in the bird species richness and abundance with different height canopy vegetation (i.e. <3.0 m, 3.0–12.0 m, and >12.0 m) during pre- and post- storm sampling periods. Mean values (±SE) are displayed. Level of significance: *P < 0.05; **P < 0.01.

The mean perching height was 8.29 ± 0.59 m (400 records from 70 species) in pre-storm, and 4.71 ± 0.37 m (269 records from 58 species) afterwards, respectively. Although population declines (log pre/post storm) were not related to foraging height (Pearson’s Correlation; *r* = 0.225, *P* = 0.117, n = 50), there was a significant decrease in perching height (*T* = 4.557, *df* = 667, *P* < 0.001) for species present during pre- and post-storm (Table S2). Particularly, five species [Red-headed Trogon (*Harpactes erythrocephalus*), Scarlet Minivet (*Pericrocotus flammeus*), Black Bulbul (*Hypsipetes leucocephalus*), Huet’s Fulvetta (*Alcippe hueti*), and Daurian Redstart (*Phoenicurus auroreus*)] had significantly shifted to lower heights following the storm (Fig. 2a, see Table S2 for statistics).

A clear trend in bird perching substrate existed among height classes, with canopy and sub-canopy species significantly experiencing severe declines. Birds perching below 3 m height showed no change in species richness pre- and post-storm (*T* = 1.147, *df* = 4, *P* = 0.436), but lower richness was recorded after the storm for birds perching in the other two height classes (for 3−12 m, *T* = 4.123, *df* = 4, *P* = 0.015; for >12 m, *T* = 3.253, *df* = 4, *P* = 0.031; Fig. 2b). For relative abundances (Fig. 2c), there was no difference for birds perching less than or equal to 12 m height (for <3 m, *T* = 1.806, *df* = 4, *P* = 0.325; for 3–12 m, *T* = 1.544, *df* = 4, *P* = 0.371), but lower abundance in post-storm for birds perching higher than 12 m (*T* = 3.011, *df* = 4, *P* = 0.039).

### The spatial congruence of bird-habitat associations

**Figure 3:**
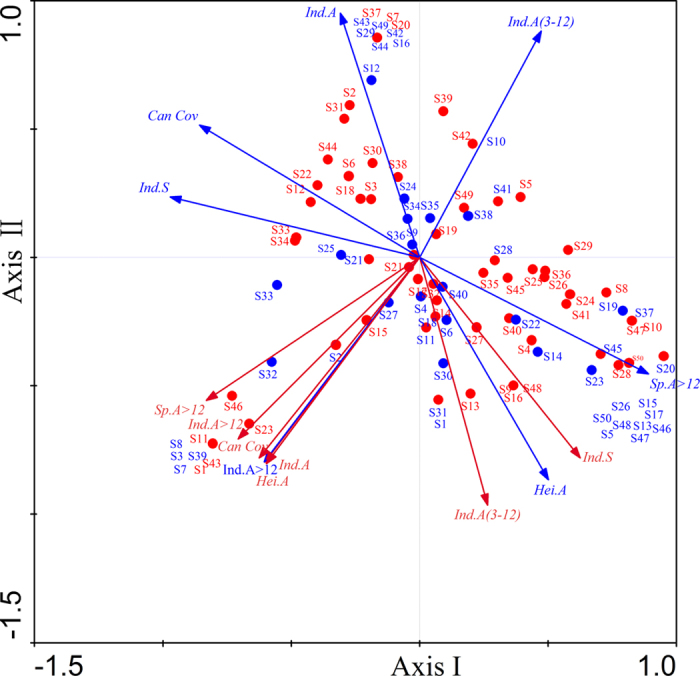
Ordination biplot of a canonical correspondence analysis (CCA) conducted on bird species as a function of vegetation variables that were highly correlated with the axes (i.e. seven pairs of variables with correlation coefficients >0.30; Table 1). Refer to Table S1 and Table 1 for abbreviation of bird species and vegetation variable codes. Red color and blue color arrows represent pre- and post- storm values, respectively.

Canonical correspondence analysis (CCA) was used to relate the 50 bird species with a set of vegetation variables (Fig. 3). The first axis (axis I) explained 24.8% of the site/species matrix variance, which is consequently associated with large trees, height of arborous species and canopy cover; while the second axis (axis II) associated with both arborous and shrub species explained 15.5% of the site/species matrix variance. Ordination indicated that two bird communities were well separated with no overlap of hull areas, and the distribution of birds associated with seven pairs of vegetation variables (Monte Carlo test, *P* < 0.05). For vegetation variables, pre-storm group was strongly dominated by larger trees (Sp.A >12, *r* = 0.91, *P* = 0.001), as indicated by the length of the vector, whereas post-storm group was dominated by shrub and sub-canopy trees (Ind.S, *r* = 0.82, *P* = 0.001).

Based on the species-level biplots and intraset correlations with vegetation vector, bird species depending on forest were largely confined to in quadrant III (lower left), and species that were frequently associated with open habitat and forest edge confined to quadrant I (upper right) during the pre-storm. For example, the former are represented by Crested Kingfisher, Black-throated Tit (*Aegithalos concinnus*), Greater Necklaced Laughingthrush (*Garrulax pectoralis*), and Orange-bellied Leafbird (*Chloropsis hardwickei*), while the latter contains Common Kingfisher (*Alcedo atthis*), Long-tailed Shrike (*Lanius schach*), Hill Prinia (*Prinia atrogularis*) and Tristram’s Bunting (*Emberiza tristrami*). However, vegetation changes following the storm resulted in some species that were located in quadrant II (upper left) in pre-storm [e.g. Chestnut Bulbul (*Hemixos castanonotus*)], and some species that used to be in quadrant IV (lower right) [e.g. Huet’s Fulvetta and Fork-tailed Sunbird (*Aethopyga christinae*)], were found in quadrant I afterwards. Similarly, some species that used to be in quadrant II [e.g. White-crowned Forktail (*Enicurus leschenaulti*) and White-rumped Munia (*Lonchura striata*)] were in quadrant III after the storm. Especially for species with significant population variation after the storm (refer to Fig. 1d), Light-vented Bulbul and Red-whiskered Bulbul shifted from quadrant II to IV, likely moving from lower to higher habitat quality. Conversely, the population decline of Crested Kingfisher (from quadrant III to IV), Blyth’s Kingfisher (IV to III) and Orange-flanked Bush Robin (IV to III) were closely associated with the disturbance and degradation of native forest structure. Meanwhile, similar patterns also existed in species with significant height changes (refer to Fig. 2a), such as Black Bulbul (II to IV), Huet’s Fulvetta(IV to I), Daurian Redstart(IV to II) and Red-headed Trogon(IV to II). Overall, the results of CCA illustrated the impacts of changes in forest structure on the bird assemblages.

## Discussion

The presence of forest destructions created by a widespread ice storm in 2008 affected the response of bird assemblages in three ways: (i) EWE-induced disturbances can affect bird assemblages depending on species-oriented characteristics/responses, and result in potential homogenization by filtering out functionally unique species. For instance, the depletion of forest-interior specialists, and enrichment of open-habitat generalists were noted; (ii) Beside the short-term population responses, community reassembly can be rapidly compensated by species displaying highly adaptive abilities, such as more mixed-species flocking social behavior, and lowering perching height; (iii) A series of changes in vegetation canopy structure and physiognomic factors following the storm have affected the composition and distribution of bird communities, which can be used as indicators to monitor and evaluate forest restoration and successional dynamics after the storm. Hence, it is essential to understand the implications of such an ecosystem disturbance on how birds response to the continuing threat of extreme weather.

### Species-specific and guild-dependent responses to the 2008 storm

Both theory and empirical studies suggest that species at higher trophic levels are more sensitive to climate change than those species at lower trophic levels^9^,^27^,^40^,^41^. Our study showed that forest specialists of several groups were adversely affected by forest modification following the storm, specifically insectivores (arboreal foliage gleaners and terrestrial insectivores), bark probers (woodpeckers), and biome-restricted species (i.e. kingfishers, dippers and barbets). This suggests that forest heterogeneity when altered substantially can filter out habitat specialists and functionally unique species following EWE. First, insectivores are considered especially vulnerable to forest modification and avoid the forest edge, possibly due to food scarcity and fragmentation-related nest predation^42^,^43^,^44^,^45^. Given adult Lepidoptera and their larvae are the most important food resources for insectivores, it may be used as an indication for changes in insectivores bird population^46^. In Nanling Mountains, Chen *et al.*^18^ and Wang *et al.*^19^ reported that population density of butterflies decreased, and many species were absent following the storm indicating a potential causal factor underlying declines in insectivorous birds. Moreover, decreased breeding success has been documented for several insectivorous specialists following forest destructions^47^,^48^,^49^,^50^. Second, bark probers (woodpeckers), frugivore–predator (barbets) and piscivores (kingfishers) also declined or became extirpated disproportionately. Since woodpeckers depend on larger trees and the proportion of damaged trees during the 2008 storm increased with DBH (those with DBH over 9.0 cm being the mostly damaged^14^,^16^), it is easy to understand why woodpeckers disappeared post storm. Blais *et al.* also found that abundances of all tree forager species [Downy Woodpecker (*P*. *pubescens*), Hairy Woodpecker (*P*. *villosus*)] decreased significantly following a storm^33^. The storm also impacted the local biogeochemical and hydrological processes^51^, for example, it covered much of the area’s shallow waterways with ice therefore prohibiting predation and also likely reduced fish and frog populations which affected the kingfishers and dippers. Indeed, one expects specialist and rare species to be doubly impacted when subjected to EWE. They are more negatively affected than generalists by habitat modifications^32^,^52^,^53^, and they tend to provide more unique yet vulnerable functions^4^,^54^,^55^. Environmental changes are filtering out specialist species and narrowing the available range of species-specific responses through the loss of unique species functions.

Besides the depletion of forest specialists, the 2008 storm seems to be responsible for both the increase of open-habitat/edge species, and more broadly functional homogenization. Changes in community composition toward enrichment in species with a specific ecological strategy (specialist vs. generalist) or functional traits have generally been treated as a response to specific habitat changes, however, only a few studies have explored the specific impact of climate change on the potential directional homogenization of assemblages^32^,^56^,^57^. In south China, the Light-vented Bulbul and Red-whiskered Bulbul are abundant species occurring in a diverse array of habitats (e.g. forest edge, plantations, farmland and other human dominated areas), and foraging in trees/bushes and feeding on a wide variety of fruits, seeds, plant matter and insects^44^. Studies have shown that the abundance of the ecologically similar European Starling (*Sturnus vulgaris*) and Dark-eyed Junco (*Junco hyemalis*) increased at affected sites in the year following an ice storm event in North America ^26,33^. A wide range of diet and habitat flexibility of these species may play a key role in their increase in abundance following a disturbance event. For instance, the Light-vented Bulbul was historically distributed to south of the Yangtze River, but has recently exhibited a strong northward expansion^58^. Extreme disturbance likely favor invasive species, ecological opportunists and species adapted to disturbed environments, particularly those adapted to human dominated environments^4^,^9^,^59^,^60^. These changes correspond to a nonrandom reconfiguration and homogenization in species compositions: habitat generalists and species with fewer specialized adaptations are those whose populations seem to benefit the most from the impacts of severe storms.

### Plasticity of bird social behavior and perch height in response to EWE

Biotic interactions are among the most important forces structuring ecological communities and are commonly climate-dependent^61^,^62^. EWEs are likely to trigger ecosystem-level disturbances, and may affect interspecific organization and functional attributes of entire ecosystems^9^,^28^,^37^,^63^. Mixed-species flocks are a common social animal organization that is susceptible to habitat degradation and human disturbance in south China^64^. Although bird diversity was severely affected by the storm event, one of the most puzzling findings of the present study was that the propensity of flocking participants showed a marginal increase following the storm. In Chebaling, mixed flocks typically form around a gregarious “nuclear” species (i.e. Huet’s Fulvetta) that is attended to by several solitary or conspecific facultative species. While flocking propensity of understory insectivores were resistant to the storm event, specific foraging guilds, such as canopy and mid-story facultative species (e.g. minivet, yuhina and flowerpecker) were more inclined to participate in flocks led by Huet’s Fulvetta (Fig. 1f, Table S1). The adaptation of social flocking strategies following the 2008 storm may provide support for the two hypothesized benefits for flock participations, including anti-predator vigilance and increasing foraging efficiency^65^,^66^,^67^.

Perching height also shows a clear trend, with canopy and sub-canopy species significantly shifting downwards to lower layers. Range shifts imply that species might be forced to interact with those from which they were formerly not overlapped. Climate-induced species’ range shifts have been reported along altitudinal^68^,^69^,^70^,^71^ and latitudinal gradients^72^,^73^,^74^,^75^,^76^. Other studies demonstrated a similar response in avian perch height following a weather-related disturbance. Cerulean Warblers (*Dendroica cerulea*) for example were shown to adjust nest height according to vegetation alteration after an storm^77^. In this study, the observed shift to lower post-disturbance foraging substrates may be interpreted as a result of nest predation by raptors and vegetation destruction. However, too little rigorous study of nest predation rates exists for EWE, mostly due to the difficulty of successfully finding and monitoring a sufficient number of nests. Much of what we do know comes from fragmented forests, in which opening and gaps makes birds more likely to be depredated^43^,^48^,^78^,^79^. Therefore, although indirect impacts may affect the community negatively in some species, others may show adaptive resilience to EWE and rapidly compensated by displaying highly plastic behavioral strategies.

### The potential impacts of the 2008 storm on local assemblages indirectly caused by vegetation modifications

Species behavior and distribution are not isolated processes; they are connected through interactions with other species and through the indirect effects of habitat modifications. Pre- and post- vegetation measurements in this study revealed that the prevalence of larger trees decreased significantly following the storm. At the most severely damaged Nanling areas, forest structure, measured in terms of vegetation composition and canopy cover, also significantly changed as a result of the storm^15^; trees with a DBH below 9.0 cm were mostly “broken off” while those over 9.0 cm were mostly “beheaded (tree crowns were removed)” during the storm^14^. Therefore, the decreased canopy bird diversity and perching height were closely associated with storm-induced changes in vegetation heterogeneity.

CCA provided a quantitative assessment of the changes in bird assemblages with reference to specific ecological strategies and/or functional traits, which have generally been treated as a response to specific habitat changes. Few species recorded between pre- and post-storm occurred within the same quadrant (i.e., no change), which may be due to drastic shifts in vegetation structure that probably directly affected bird assemblage patterns. Specialists within higher trophic levels are more sensitive to extirpation or decline due to the combined impact of habitat modifications and severe disturbance. Previous studies have indicated that forest-dependent insectivores (i.e. arboreal foliage gleaners, understory foragers and bark probers) were closely associated with a high proportion of native canopy cover^44^. Brandt *et al.* also found that high canopy cover and large trees were keystone structures whose presence added resource heterogeneity, thereby facilitating greater species richness^80^. At a landscape scale, the 2008 storm may have substantially increased the amount of edge habitat and forest gaps, and as a result inhibited the survival and colonization of those insectivores.

By contrast, species associated with open-habitat and forest edge were prone to benefit from EWE disturbance. All these species that are positively associated with dense shrub-cover, often are found nesting among stumps, up-turned root-balls, and downed limbs or logs, and hence are less affected by loss of canopy cover. These species may be taking advantage of a unique combination of resources that occurs after an extreme storm event, including food and the presence of live foliage to conceal nests^34^. In fact, the effects of EWE and habitat change can be jointly assessed within a given biogeographical region. Studies have reported impacts of fires at the community level are comparable to accumulated climate changes by shifting communities towards early succession species^11^. Similarly, hurricane-induced changes in forest habitat and the use of refuges by birds displaced from hurricane-damaged forests have comparable impacts^81^. Hence, a series of changes in canopy structure and vegetation heterogeneity variables following the storm directly affected the composition and distribution of bird communities. Findings from the present study also indicated that bird assemblages with specific ecological strategies and functional traits, could serve as indicators to characterize the ecosystems they inhabit, thus being particularly suitable for monitoring forest regeneration and successional dynamics following extreme weather events.

## Conclusions

In the context of the ongoing biodiversity crisis and EWE frequency/intensity, identifying species that are particularly vulnerable to disturbance, and traits that predispose them to vulnerability, is crucial to strategically plan conservation initiatives. The BACI approach employed before and after the 2008 storm in China, combined with our attention to mixed-species flocking behaviour and perching height, revealed assembly patterns that were consistent for both vegetation and bird communities. Our analysis shows that species functional traits (e.g. habitat preference, feeding group and flocking plasticity), and forest heterogeneities (e.g. intact canopy cover and high density of large trees) interact synergistically to influence a species’ response to the storm at the local scale. Beyond the traits used in our study, other factors such as the fine-scale division of resources and space (e.g. morphological measurements), and life-history traits (e.g. adult mass, foraging and nesting strategies, and dispersal ability) could also contribute to reassemble community structure. Furthermore, since species interactions can dramatically alter species responses to EWE, a more complex network approach might become necessary that will require scaling from modules to entire food webs. Overall, we suggest that generating accurate predictions of responses to EWE will be crucial for conserving natural and human-influenced ecosystems, particularly if a combined long-term monitoring framework that highlights species-oriented characteristics and behavioral adaptations, across spatial-temporal scales and habitat variations is employed.

## Methods

### Study site

The study was conducted at Chebaling National Natural Reserve located in Guangdong province, southern China (24°40′-24°46′N, 114°09′-114°16′ E). The Reserve protects 7545 ha of subtropical evergreen broadleaf forests and rare flora and fauna. As a boundary area between tropical and subtropical flora, Chebaling is an important focal point of the Nanling mountain hotspot ecoregions^82^. The region has served as a refuge for biodiversity since the Quaternary, including several biotic ecoregions with many endemic species, and it has played a major role in the evolution of the terrestrial biota of south China^83^,^84^,^85^. The climate is typical subtropical monsoon climate, with an annual average temperature of 19.6 °C and long-term average annual rainfall of 1468 mm. In the reserve, a total of 1928 plant species and 1558 animal species have been identified and documented^86^.

A destructive ice storm event occurred in southern China mostly from 25 January until 6 February 2008. After that, unusually low temperatures continued for two weeks, and the mean temperature of February 2008 was lower than before and after the year (Fig. S1). Among the hardest hit areas was the forest ecosystem of Chebaling at Nanling Mountain which was severely damaged by a freezing rain and ice accumulation. Forest destruction between 500 and 1300 meters in elevation at Nanling, north Guangdong was quantified. There were four observed types of damage to the trees, which including being “broken off”, “bending”, “lodging” and “beheaded”. Most damaged trees were “broken off”, accounting for 88% of the total in the Slash Pine *Pinus elliottii* stand, and 64% in the broadleaved stand^14^. Su *et al.* reported that tree damage differed with species and DBH size classes, with the most severely damaged tree members of Fagaceae in Chebaling^16^. The trees with a DBH below 9.0 cm were mostly “broken off” while those with DBH over 9.0 cm were mostly “beheaded”^14^.

### Bird and vegetation surveys

Based our fixed monthly monitoring activities from 2007 to 2009 at Chebaling, the 2008 ice storm presented an unique opportunity to study the effects of EWE-induced damages on bird reassembly. A before–after/control–impact (BACI) method^39^ in which both bird community and vegetation characteristics were simultaneously measured at independent sites was used to assess the forest community and structure. Bird assemblages were monthly censused repeatedly both before (from Jan to Dec 2007) and after (from March 2008 to March 2009) the storm event. Using fixed point counts and line transects, points were established along three preexisting trails, with each trail consisting of 10 sampling points at least 250 m from each other^87^. The censuses were initiated at sunrise and terminated before 10:30 a.m. on windless and rainless days. Within a 25 m radius plot, two observers simultaneously detected all birds by either visual or auditory detections lasting 10 min. We also recorded foraging height (when seen) and activity (i.e. calling, flying, perching, foraging, nesting, and flocking) of all individuals^44^,^64^,^67^, which permitted us to directly evaluate whether vegetation structural changes caused by the storm affected bird behavior, such as foraging strategy.

Vegetation structures were first sampled within quadrats (10 × 10 m) around each bird count points at April and October during 2007, and replications were made at the same month during 2008. In each quadrat, the following variables were measured by a botanist, and recorded by another simultaneously: (i) DBH and height of all arborous tree species (woody plants with DBH >1 cm, defined as 1.3 m); (ii) percentage of tree canopy cover (Can Cov), using a spherical densitometer, following Zhang *et al.*^44^; (iii) percentage of shrub cover (Shr Cov, woody plants with DBH <1 cm) and percent herb cover (Her Cov, non-woody plants with height<1 m); (iv) abundance of dead trees (NTD); (v) leaf litter depth (LLD, average of 10 readings from 10 random locations using a ruler). For statistical purposes, the vertical distribution of tree species was assigned to one of three classes: <3.0 m, 3.0–12.0 m, and >12.0 m. The quadrat surveys were conducted at least 10 m away from the birding transects and points. Although vegetation sampling plots were of 10 m × 10 m quadrat, we assumed that they correctly reflect vegetation structure within the bird count points of 25 m radius.

### Species characterizations by different functional traits

To facilitate comparing avifaunal changes before and after the storm, species assignments to ecological categories/functional guild were based on the datasets of Zhao^88^ and Zhang *et al.*^44^,^55^, and with updates based on prior field experience. Each species was assigned to one mutually exclusive category with respect to five ecological characteristics: habitat preference, dietary guild, social flocking, migratory status and human tolerance. According to their primary use of habitat type and structure for nesting and movement, species were first categorized into one of five habitat preferences: “edge species (ES)”; “edge-tolerant forest species (ETF)”; forest specialist (FS)”; “generalist (G)”; “open-habitat species (OS)”. Species were then allocated one of ten dietary guilds: “arborous foliage glean insectivore (AFGI)”; “arborous foliage glean insectivore-frugivore (AFGIF)”; “arboreal frugivore–predator (AFP)”; “bark-gleaning insectivore (BGI)”; “miscellaneous insectivore-piscivore (MIP)”; “nectarivores-insectivore-frugivore (NIF)”; “sallying insectivore (SI)”; “terrestrial frugivore (TF)”; “terrestrial insectivore (TI)”; “terrestrial insectivore-frugivore (TIF)”. Species were also assigned to one of four categories of migration status: “permanent resident (R)”, “winter visitor (W)”, “summer visitor (S)”, and “passage migrant (P)”. Flocking guilds consisted of three categories: “Non-flocking species (NON)”; “monospecific flock participants (MONO)” and “mixed-species flock participants (FLOCK)”. Human tolerance consisted of three categories: “vulnerable to human disturbance (VUL)”; “resistant to human disturbance (RES)” and “moderate susceptibility (MODER)”. For a complete list of species with functional traits and data, see Supplementary Information (Table S1).

### Data analysis

All data sets of variables were examined for normality using the Kolmogorov–Smirnov Test. Then non-parametric Wilcoxon Signed Ranks Test were used for Two-Related-Sample concerning vegetation characteristics data; while Paired T-Test was used to analyze differences in relative abundance of bird functional-guild with STATISTICA 7.1 (StatSoft. inc, Tulsa, USA). To compare bird assemblages between pre- and post-storm, we first assessed whether our sampling effort was sufficient to represent the species richness of each period. Rarefaction analysis was used to compare rates of species accumulation for point count data based on a Monte Carlo simulation procedure (1000 runs) implemented with EcoSim 7.2 (Acquired Intelligence & Kesey-Bear Inc, Vermont, USA). Secondly, an estimate of the “true” species richness was calculated by EstimateS Win 8.2.0 (University of Connecticut, Storrs, USA), using the mean of the nine commonly employed nonparametric estimators: ACE, ICE, Chao1, Chao2, Jack1, Jack2, Bootstrap, MMruns and MMmeans.

At the transect level, regression plots obtained from Generalized Linear Model Fit were used to identify the storm severity that affected bird population fluctuation (e.g. rare species vs. common species, variables were log-transformed [log (x + 1)] and down weighted for rare species and dominant species), while simultaneously controlling for potential spatial non-independence of transects with sampling point nested in pre vs. post storm as a random effect. For every species, we calculated the mean number of individuals counted at each sampling point, and compared population changes in response to the storm. We also constructed a guild matrix by counting the number of species in each guild (species that were recorded only once were excluded), especially for habitat preference, dietary guild, flocking behaviour, migration status and human tolerance, which was used to test whether bird population declines were affected by specific ecological or functional traits. Similarly, perching height and substrate were also compared to examine niche shift/behavioral adaptations of bird species, and the indirect effect of the storm on canopy vegetation structure. Flocking propensity of a species was defined as the percentage of individuals in flocks divided by the total number of foraging birds sighted in all point counts^64^. All statistical analyses were carried out using STATISTICA 7.1, and all figures were plotted using OriginPro8.SR3.

To determine the response of forest birds with specific ecological strategy or functional traits to the widespread changes in vegetation structure following the storm, we used multivariate analyses to relate the data on bird abundances at the points to vegetation variables in CANOCO 4.5 (Biometris–Plant Research International, Wageningen, Netherlands). A preliminary detrended correspondence analysis (DCA) showed a maximum gradient length of 6.931 for pre-storm and 7.596 for post-storm, which suggested a unimodal distribution, and required a canonical correspondence analysis (CCA). CCA can be represented by joint biplots of the species and site ordination scores in which quantitative environmental variables are depicted as arrows (i.e. distance and angle of species scores from the center point on the plot indicate strength of environmental preferences) and binary environmental variables are shown as centroids. The significance of the CCA ordination of species–habitat relationships was investigated by performing a randomization test on the predicted relationships (Monte Carlo Random Permutations, n = 499). To optimize the representation of species, axis scores were rescaled using inter-species distances and biplot-scaling.

## Additional Information

**How to cite this article**: Zhang, Q. *et al.* Avian responses to an extreme ice storm are determined by a combination of functional traits, behavioural adaptations and habitat modifications. *Sci. Rep.* **6**, 22344; doi: 10.1038/srep22344 (2016).

## Supplementary Material

Supplementary Information

## Figures and Tables

**Table 1:** Mean values (±SE) for vegetation variables, and result of pre- and post-storm comparisons at Chebaling, Southern China.

Vegetation variables	Code	Pre-storm	Post-storm	Z	P
No. arborous species	Sp. A	9.0 ± 1.0	6.6 ± 0.9	−1.829	0.067
No. arborous species<3 m	Sp. A <3	2.4 ± 0.5	1.4 ± 0.6	−1.510	0.131
No. arborous species = 3–12 m	Sp. A (3–12)	8.4 ± 1.5	6.2 ± 0.9	−0.420	0.674
No. arborous species >12 m	Sp. A >12	4.7 ± 0.9	1.3 ± 0.5	−2.388	0.017*
No. arborous individuals	Ind. A	19.9 ± 2.7	13.3 ± 1.4	−1.863	0.063
No. arborous individuals<3 m	Ind. A <3	3.5 ± 1.1	1.6 ± 0.8	−1.511	0.131
No. arborous individuals 3–12 m	Ind. A (3–12)	10.8 ± 2.2	9.5 ± 1.9	−1.053	0.292
No. arborous individuals >12 m	Ind. A >12	5.6 ± 1.8	2.1 ± 0.4	−2.207	0.027*
DBH of arborous (cm)	DBH. A	24.6 ± 1.6	27.0 ± 2.0	−0.028	0.978
Height of arborous (m)	Hei. A	10.5 ± 0.6	8.5 ± 0.9	−3.646	0.001**
Canopy cover of arborous	Can Cov	0.8 ± 0.1	0.7 ± 0.1	−0.957	0.339
No. shrub species	Sp. S	6.3 ± 0.9	8.4 ± 1.1	−1.612	0.107
No. shrub individuals	Ind. S	19.5 ± 4.3	27.8 ± 7.6	−1.332	0.183
Height of shrub (m)	Hei. S	0.8 ± 0.1	0.9 ± 0.1	−0.028	0.977
Shrub cover	Shr Cov	0.4 ± 0.1	0.5 ± 0.1	−0.021	0.656
No. grass species	Sp. G	3.9 ± 1.5	5.4 ± 0.5	−1.903	0.057
Grass cover	Gra Cov.	50.3 ± 12.6	62.4 ± 12.5	−1.122	0.262
No. dead trees	DEAD	1.25 ± 0.05	2.43 ± 0.07	−1.036	0.304
Leaf litter depth	LLD	3.30 ± 0.31	5.52 ± 0.63	−0.530	0.586

The Z values are derived from Wilcoxon Signed Ranks Test. Level of significance: *P < 0.05; **P < 0.01.

## References

[b1] Easterling D. R. *et al.* Climate extremes: observations, modeling, and impacts. Science. 289, 2068–2074 (2000). 11000103 10.1126/science.289.5487.2068

[b2] Walther G.-R. *et al.* Ecological responses to recent climate change. Nature. 416, 389–395 (2002). 11919621 10.1038/416389a

[b3] Parmesan C. Ecological and evolutionary responses to recent climate change. Annu. Rev. Ecol. Evol. S. 37, 637–669 (2006).

[b4] Mouillot D., Graham N. A., Villéger S., Mason N. W. & Bellwood D. R. A functional approach reveals community responses to disturbances. Trends. Ecol. Evol. 28, 167–177 (2013). 23141923 10.1016/j.tree.2012.10.004

[b5] Easterling D. R. *et al.* Observed Variability and Trends in Extreme Climate Events: A Brief Review*. B. Am. Meteorol. Soc. 81, 417–425 (2000).

[b6] Jiguet F., Gadot A.-S., Julliard R., Newson S. E. & Couvet D. Climate envelope, life history traits and the resilience of birds facing global change. Global. Change. Biol. 13, 1672–1684 (2007).

[b7] Lavergne S., Mouquet N., Thuiller W. & Ronce O. Biodiversity and climate change: integrating evolutionary and ecological responses of species and communities. Annu. Rev. Ecol. Evol. S. 41, 321–350 (2010).

[b8] Pearson R. G. & Dawson T. P. Predicting the impacts of climate change on the distribution of species: are bioclimate envelope models useful? Global. Ecol. Biogeogr. 12, 361–371 (2003).

[b9] Gilman S. E., Urban M. C., Tewksbury J., Gilchrist G. W. & Holt R. D. A framework for community interactions under climate change. Trends. Ecol. Evol. 25, 325–331 (2010). 20392517 10.1016/j.tree.2010.03.002

[b10] Rouault G. *et al.* Effects of drought and heat on forest insect populations in relation to the 2003 drought in Western Europe. Ann. Forest. Sci. 63, 613–624 (2006).

[b11] Clavero M., Villero D. & Brotons L. Climate change or land use dynamics: do we know what climate change indicators indicate? PLoS ONE. 6, e18581 (2011). 21533025 10.1371/journal.pone.0018581 PMC3080866

[b12] Stone R. Ecologists report huge storm losses in China’s forests. Science. 319, 1318–1319 (2008). 18323420 10.1126/science.319.5868.1318

[b13] Corlett R. T. The ecology of tropical East Asia. (Oxford University Press, 2014).

[b14] Chen H. *et al.* Comparison between characteristics of ice damage to three stands. J. South. China. Agr. Univ. 31, 78–81 (2010) (in Chinese with English abstract).

[b15] He Q. *et al.* Types and extent of damage to Cunninghamia lanceolata plantations due to unusually heavy snow and ice in southern China. Chin. J. Plant. Ecol. 34, 195–203 (2010) (in Chinese with English abstract).

[b16] Su Z., Liu G., Ou Y., Dai Z. & Li Z. Storm damage in a montane evergreen broadleaved forest of Chebaling National Nature Reserve, South China. Chin. J. Plant. Ecol. 34, 213–222 (2010) (in Chinese with English abstract).

[b17] Song H. Survey of destruction due to the snow calamity on the ecological environment of the south China nature reserves. Chin. J. Wildl. 29, 147–149 (2008) (in Chinese with English abstract).

[b18] Chen R. *et al.* Effect of the 2008 ice storm on the butterfly resources in Nanling area of China. J. Ecol. Sci. 27, 478–482 (2008) (in Chinese with English abstract).

[b19] Wang S., Gong Y., Gu D., Kang L. & Xu D. Pieridae fauna of Nanling National Nature Reserve and the impact of snow disaster on the population densities. Sci. Silvae. Sinicae. 44, 184–187 (2008) (in Chinese with English abstract).

[b20] Li Z., Li L., Zhang D. & Yu Q. Influence of prolonged low temperature, snowfall and freezing rain on birds in Liangzi Lake Basin in 2008. Chin. J. Wildl. 29, 290–293 (2008) (in Chinese with English abstract).

[b21] Su H. *et al.* Population dynamics of the Golden Pheasant (*Chrysolophus pictus*) in the Three Gorges Reservoir Area after the snow calamity in 2008. Sci. Silvae. Sinicae. 44, 75–81 (2008) (in Chinese with English abstract).

[b22] Cheng S. Ecological Effect of Ice-snow Frozen Disaster on Silver Pheasant Population in Wuyi Mountain Nature Reserve. Chin. J. Wildl. 30, 314–316 (2009) (in Chinese with English abstract).

[b23] Ou Y., Su Z., Li Z., Tong F. & Liu Z. Soil arthropod diversity following an ice storm in a montane evergreen broadleaved forest in Chebaling National Nature Reserve, China. Biodiv. Sci. 17, 440–447 (2009) (in Chinese with English abstract).

[b24] Brook B. W., Sodhi N. S. & Bradshaw C. J. Synergies among extinction drivers under global change. Trends. Ecol. Evol. 23, 453–460 (2008). 18582986 10.1016/j.tree.2008.03.011

[b25] Van De Pol M. *et al.* Do changes in the frequency, magnitude and timing of extreme climatic events threaten the population viability of coastal birds? J. Appl. Ecol. 47, 720–730 (2010).

[b26] Faccio S. D. Effects of ice storm-created gaps on forest breeding bird communities in central Vermont. Forest. Ecol. Manag. 186, 133–145 (2003).

[b27] Voigt W. *et al.* Trophic levels are differentially sensitive to climate. Ecology. 84, 2444–2453 (2003).

[b28] Walther G.-R. Community and ecosystem responses to recent climate change. Philos. T. R. Soc. B. 365, 2019–2024 (2010). 10.1098/rstb.2010.0021 PMC2880129 20513710

[b29] Jiguet F., Brotons L. & Devictor V. Community responses to extreme climatic conditions. Curr. Zool. 57, 406–413 (2011).

[b30] McKinney M. L. & Lockwood J. L. Biotic homogenization: a few winners replacing many losers in the next mass extinction. Trends. Ecol. Evol. 14, 450–453 (1999). 10511724 10.1016/s0169-5347(99)01679-1

[b31] Stachowicz J. J., Terwin J. R., Whitlatch R. B. & Osman R. W. Linking climate change and biological invasions: ocean warming facilitates nonindigenous species invasions. P. Natl. Acad. Sci. USA 99, 15497–15500 (2002). 10.1073/pnas.242437499 PMC137745 12422019

[b32] Gaüzère P., Jiguet F. & Devictor V. Rapid adjustment of bird community compositions to local climatic variations and its functional consequences. Global. Change. Biol. 10.1111/gcb.12917 (2015). 25731935

[b33] Blais J., Savard J.-P. L. & Gauthier J. Impact of an ice storm on resident bird populations in eastern North America. For. Chro. 77, 661–666 (2001).

[b34] Smucker K. M., Hutto R. L. & Steele B. M. Changes in bird abundance after wildfire: importance of fire severity and time since fire. Ecol Appl. 15, 1535–1549 (2005).

[b35] Thibault K. M. & Brown J. H. Impact of an extreme climatic event on community assembly. P. Natl. Acad. Sci. USA 105, 3410–3415 (2008). 10.1073/pnas.0712282105 PMC2265133 18303115

[b36] Du Y., Mi X., Liu X. & Ma K. The effects of ice storm on seed rain and seed limitation in an evergreen broad-leaved forest in east China. Acta Oecol. 39, 87–93 (2012).

[b37] Parmesan C., Root T. L. & Willig M. R. Impacts of extreme weather and climate on terrestrial biota*. B. Am. Meteorol. Soc. 81, 443–450 (2000).

[b38] Lande R. Adaptation to an extraordinary environment by evolution of phenotypic plasticity and genetic assimilation. J. Evolution. Biol. 22, 1435–1446 (2009). 10.1111/j.1420-9101.2009.01754.x 19467134

[b39] Stewart-Oaten A., Murdoch W. W. & Parker K. R. Environmental impact assessment:” Pseudoreplication” in time? Ecology. 67, 929–940 (1986).

[b40] Vasseur D. A. & McCann K. S. A Mechanistic Approach for Modeling Temperature‐Dependent Consumer‐Resource Dynamics. Am. Nat. 166, 184–198 (2005). 16032573 10.1086/431285

[b41] Zarnetske P. L., Skelly D. K. & Urban M. C. Biotic multipliers of climate change. Science. 336, 1516–1518 (2012). 22723403 10.1126/science.1222732

[b42] Laurance W. F. *et al.* Ecosystem decay of Amazonian forest fragments: a 22‐year investigation. Conserv. Biol. 16, 605–618 (2002).

[b43] Sekercioglu C. H. *et al.* Disappearance of insectivorous birds from tropical forest fragments. P. Natl. Acad. Sci. USA 99, 263 (2002). 10.1073/pnas.012616199 PMC117549 11782549

[b44] Zhang Q., Han R. C. & Zou F. S. Effects of artificial afforestation and successional stage on a lowland forest bird community in southern China. Forest. Ecol. Manag 261, 1738–1749 (2011).

[b45] Powell L. L., Cordeiro N. J. & Stratford J. A. Ecology and conservation of avian insectivores of the rainforest understory: A pantropical perspective. Biol. Conserv. 188, 1–20 (2015).

[b46] Barbaro L. & Van Halder I. Linking bird, carabid beetle and butterfly life‐history traits to habitat fragmentation in mosaic landscapes. Ecography. 32, 321–333 (2009).

[b47] Jones J., DeBruyn R. D., Barg J. J. & Robertson R. J. Assessing the effects of natural disturbance on a Neotropical migrant songbird. Ecology. 82, 2628–2635 (2001).

[b48] Stephens S. E., Koons D. N., Rotella J. J. & Willey D. W. Effects of habitat fragmentation on avian nesting success: a review of the evidence at multiple spatial scales. Biol. Conserv. 115, 101–110 (2004).

[b49] Bolger D. T., Patten M. A. & Bostock D. C. Avian reproductive failure in response to an extreme climatic event. Oecologia. 142, 398–406 (2005). 15549403 10.1007/s00442-004-1734-9

[b50] Decker, K. L. & Conway C. J. Effects of an unseasonable snowstorm on Red-faced Warbler nesting success. Condor. 111, 392–395 (2009).

[b51] Lindenmayer D. *et al.* Salvage harvesting policies after natural disturbance. Science. 303, 1303 (2004). 14988539 10.1126/science.1093438

[b52] Devictor V. & Robert A. Measuring community responses to large‐scale disturbance in conservation biogeography. Divers Distrib. 15, 122–130 (2009).

[b53] Clavel J., Julliard R. & Devictor V. Worldwide decline of specialist species: toward a global functional homogenization? Front Ecol Environ. 9, 222–228 (2010).

[b54] Calba S., Maris V. & Devictor V. Measuring and explaining large‐scale distribution of functional and phylogenetic diversity in birds: separating ecological drivers from methodological choices. Global. Ecol. Biogeogr. 23, 669–678 (2014).

[b55] Zhang Q. *et al.* Do Bird Assemblages Predict Susceptibility by E-Waste Pollution? A Comparative Study Based on Species-and Guild-Dependent Responses in China Agroecosystems. PloS ONE. 10, e0122264 (2015). 25811881 10.1371/journal.pone.0122264 PMC4374810

[b56] Davey C. M., Chamberlain D. E., Newson S. E., Noble D. G. & Johnston A. Rise of the generalists: evidence for climate driven homogenization in avian communities. Global. Ecol. Biogeogr. 21, 568–578 (2012).

[b57] Le Viol I. *et al.* More and more generalists: two decades of changes in the European avifauna. Biol Letters. 8, 780–782 (2012). 10.1098/rsbl.2012.0496 PMC3441008 22809721

[b58] Xing X. Y., Alström P., Yang X. J. & Lei F. M. Recent northward range expansion promotes song evolution in a passerine bird, the Light‐vented Bulbul. J. Evolution. Biol. 26, 867–877 (2013). 10.1111/jeb.12101 23438018

[b59] Sax D. F. *et al.* Ecological and evolutionary insights from species invasions. Trends. Ecol. Evol. 22, 465–471 (2007). 17640765 10.1016/j.tree.2007.06.009

[b60] Burivalova Z., Şekercioğlu Ç. H. & Koh L. P. Thresholds of logging intensity to maintain tropical forest biodiversity. Curr. Biol. 24, 1893–1898 (2014). 25088557 10.1016/j.cub.2014.06.065

[b61] Dunson W. A. & Travis J. The role of abiotic factors in community organization. Am. Nat. 138, 1067–1091 (1991).

[b62] Tylianakis J. M., Didham R. K., Bascompte J. & Wardle D. A. Global change and species interactions in terrestrial ecosystems. Ecol. Lett. 11, 1351–1363 (2008). 19062363 10.1111/j.1461-0248.2008.01250.x

[b63] Araújo M. B. & Luoto M. The importance of biotic interactions for modelling species distributions under climate change. Global. Ecol. Biogeogr 16, 743–753 (2007).

[b64] Zhang Q., Han R. C., Huang Z. L. & Zou F. S. Linking vegetation structure and bird organization: response of mixed-species bird flocks to forest succession in subtropical China. Biodivers. Conserv. 22, 1965–1989 (2013).

[b65] Powell G. Sociobiology and adaptive significance of interspecific foraging flocks in the Neotropics. Ornithol. Monogr. 36, 713–732 (1985).

[b66] Greenberg R. Birds of many feathers: the formation and structure of mixed species flocks of forest birds : In On the Move: How and Why Animals Travel in Groups(Ed. by Boinski S. & Gerber P. A. ), pp. 521–588 (University of Chicago Press, 2001).

[b67] Zou F. S., Chen G. Z., Yang Q. F. & Fellowes J. R. Composition of mixed-species flocks and shifts in foraging location of flocking species on Hainan Island, China. Ibis. 153, 269–278 (2011).

[b68] Carroll A. L., Taylor S. W., Régnière J. & Safranyik L. Effect of climate change on range expansion by the mountain pine beetle in British Columbia. Pages 223–232 In Shore T. L. *et al.* (eds) Mountain Pine Beetle Symposium: Challenges and Solutions, Oct. 30-31, 2003. Kelowna BC. Natural Resources Canada, Infromation Report BC-X-399, Victoria (2003).

[b69] Pauli H., Gottfried M., Reiter K., Klettner C. & Grabherr G. Signals of range expansions and contractions of vascular plants in the high Alps: observations (1994–2004) at the GLORIA* master site Schrankogel, Tyrol, Austria. Global. Change. Biol. 13, 147–156 (2007).

[b70] Holzinger B., Hülber K., Camenisch M. & Grabherr G. Changes in plant species richness over the last century in the eastern Swiss Alps: elevational gradient, bedrock effects and migration rates. Plant Ecol. 195, 179–196 (2008).

[b71] Sekercioglu C. H., Schneider S. H., Fay J. P. & Loarie S. R. Climate change, elevational range shifts, and bird extinctions. Conserv. Biol. 22, 140–150 (2008). 18254859 10.1111/j.1523-1739.2007.00852.x

[b72] Parmesan C. & Yohe G. A globally coherent fingerprint of climate change impacts across natural systems. Nature. 421, 37–42 (2003). 12511946 10.1038/nature01286

[b73] Walther G.-R., Berger S. & Sykes M. T. An ecological ‘footprint’of climate change. P. Roy. Soc. B-Biol. Sci. 272, 1427–1432 (2005). 10.1098/rspb.2005.3119 PMC1559830 16011916

[b74] Lemoine N. & Schaefer H. C. & Böhning‐Gaese, K Species richness of migratory birds is influenced by global climate change. Global. Ecol. Biogeogr. 16, 55–64 (2007).

[b75] Parmesan C. Influences of species, latitudes and methodologies on estimates of phenological response to global warming. Global. Change. Biol. 13, 1860–1872 (2007).

[b76] Netherer S. & Schopf A. Potential effects of climate change on insect herbivores in European forests‒general aspects and the pine processionary moth as specific example. Forest. Ecol. Manag. 259, 831–838 (2010).

[b77] Jones J. *et al.* Minimum estimates of survival and population growth for Cerulean Warblers (*Dendroica cerulea*) breeding in Ontario, Canada. Auk. 121, 15–22 (2004).

[b78] Robinson W. D., Styrsky J. N., Brawn J. D. & Stouffer P. Are artificial bird nests effective surrogates for estimating predation on real bird nests? A test with tropical birds. Auk. 122, 843–852 (2005).

[b79] Sigel B. J., Douglas Robinson W. & Sherry T. W. Comparing bird community responses to forest fragmentation in two lowland Central American reserves. Biol. Conserv. 143, 340–350 (2010).

[b80] Brandt J. S. *et al.* Sacred forests are keystone structures for forest bird conservation in southwest China’s Himalayan mountains. Biol. Conserv. 166, 34–42 (2013).

[b81] Rittenhouse C. D. *et al.* Avifauna response to hurricanes: regional changes in community similarity. Global. Change. Biol. 16, 905–917 (2010).

[b82] Tang Z., Wang Z., Zheng C. & Fang J. Biodiversity in China’s mountains. Front Ecol Environ. 4, 347–352 (2006).

[b83] Qian H. & Ricklefs R. E. Large-scale processes and the Asian bias in species diversity of temperate plants. Nature, 407, 180–182 (2000). 11001054 10.1038/35025052

[b84] Wang J., Gao P., Kang M., Lowe A. J. & Huang H. Refugia within refugia: the case study of a canopy tree (*Eurycorymbus cavaleriei*) in subtropical China. J Biogeog. 36, 2156–2164 (2009).

[b85] Tian S., López-Pujol J., Wang H. W., Ge S. & Zhang Z. Y. Molecular evidence for glacial expansion and interglacial retreat during Quaternary climatic changes in a montane temperate pine (*Pinus kwangtungensis* Chun ex Tsiang) in southern China. Plant. Syst. Evol. 284, 219–229 (2010).

[b86] Cai D. S. & Song X. J. Bioresource and protection countermeasure in National Reserve of Chebaling in Guangdong Province. Ecol. Sci. 24, 282–285 (2005) (in Chinese with English abstract).

[b87] Bibby C. J., Burgress N. D., Hill D. A. & Mustoe S. Bird census techniques (Academic Press, 2000).

[b88] Zhao Z. J. A Handbook of the Birds of China (Jilin Science and Technology Press, 2001).

