# Supplementary material for: Avian responses to an extreme ice storm are determined by a combination of functional traits, behavioural adaptations and habitat modifications
Source: Sci Rep. 2016 Mar 1;6:22344. doi: 10.1038/srep22344 (PMC4772112; doi:10.1038/srep22344)
Supplement: Supplementary Information [file srep22344-s1.doc]

**Title:** Avian responses to an extreme ice storm are determined by a combination of functional traits, behavioural adaptations and habitat modifications

**Author list:** Qiang Zhang^1,+^, Yongmi Hong^1,+^, Fasheng Zou^1^,*, Min Zhang^1^, Tien Ming Lee^2^, Xiangjin Song^3^ & Jiteng Rao^3^


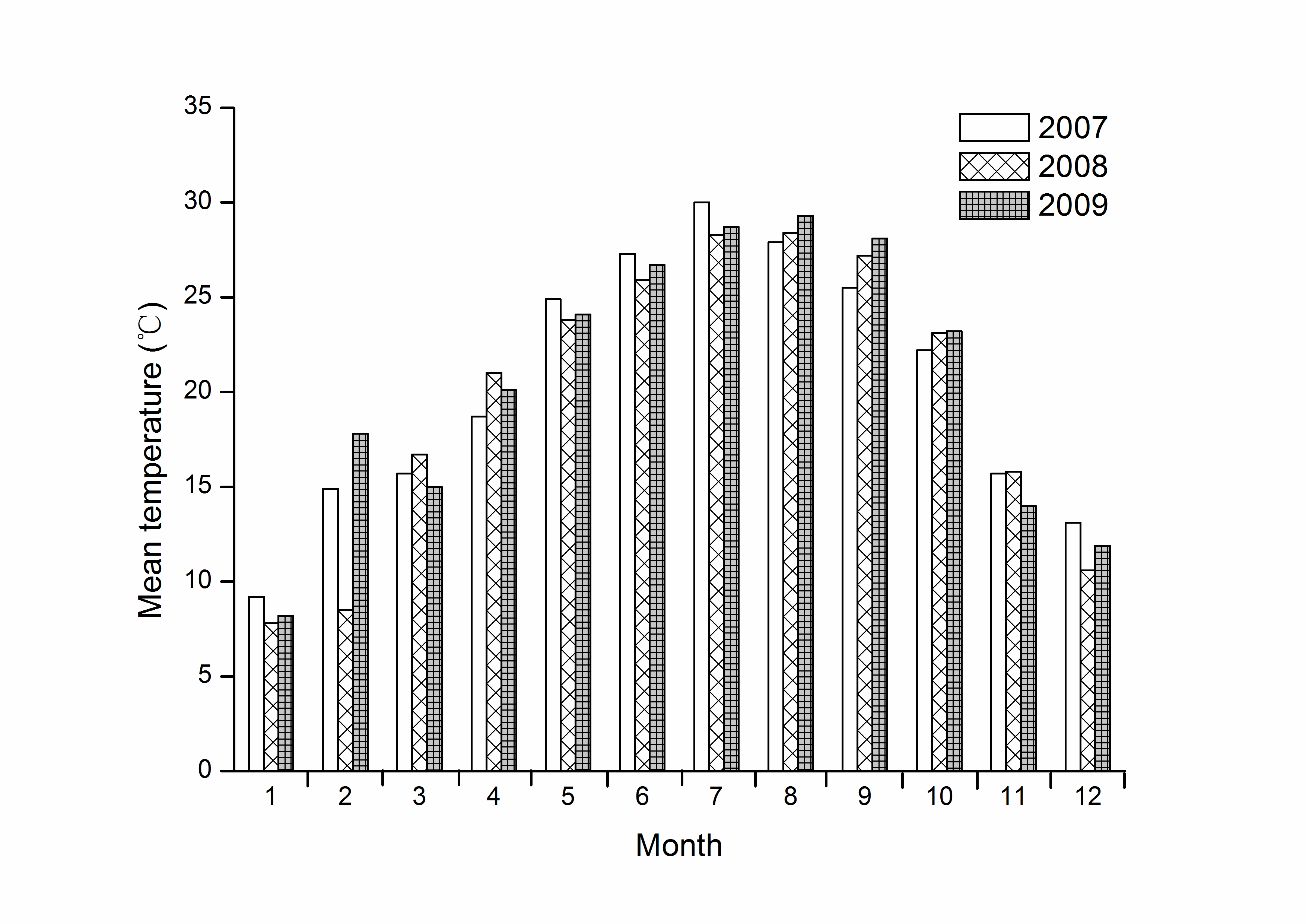
 **Figure. S1. Monthly mean temperature in Chebaling Nation Natural Reserve, Guangdong province, China from 2007-2009.**

**Table S1. Ecological or functional categorizations and encounter proportion (no of individuals/total) of all species recorded from pre- and post-storm at Chebaling, Guangdong province, China.** Data on five key ecological or functional traits (i.e. habitat preference, dietary guild, social flocking, migratory status and human tolerance) were primarily collated from Zhao (2001) and Zhang et al. (2011, 2013).

| **English name** | **Scientific name** | **Ecological/functional traits** | | | | | **Code^f^** | **Proportion** | |
| --- | --- | --- | --- | --- | --- | --- | --- | --- | --- |
|  |  | **Habitat^a^** | **Diet^b^** | **Flock^c^** | **Status^d^** | **Human^e^** |  | **Pre-storm** | **Post-storm** |
| Large Hawk-cuckoo | *Cuculus sparverioides* | ETF | AFGI | NON | S | VUL | - | 0.0003 | 0.0000 |
| Common Coucal | *Centropus sinensis* | OS | TI | NON | R | MODER | S16 | 0.0010 | 0.0004 |
| Lesser Coucal | *Centropus bengalensis* | OS | TI | NON | R | MODER | - | 0.0007 | 0.0000 |
| Red-headed Trogon | *Harpactes erythrocephalus* | FS | SI | NON | R | VUL | S25 | 0.0034 | 0.0071 |
| Dollarbird | *Eurystomus orientalis* | OS | SI | NON | S | VUL | S44 | 0.0034 | 0.0009 |
| Blyth's Kingfisher* | *Alcedo hercules* | FS | MIP | NON | R | VUL | S8 | 0.0017 | 0.0004 |
| Common Kingfisher | *Alcedo atthis* | OS | MIP | NON | R | VUL | S42 | 0.0010 | 0.0004 |
| Crested Kingfisher* | *Megaceryle lugubris* | OS | MIP | NON | R | VUL | S15 | 0.0014 | 0.0004 |
| Great Barbet | *Megalaima virens* | ETF | AFP | FLOCK | R | VUL | S13 | 0.0024 | 0.0004 |
| Black-browed Barbet | *Megalaima oorti* | ETF | AFP | FLOCK | R | VUL | - | 0.0003 | 0.0000 |
| Great Spotted Woodpecker | *Dendrocopos major* | ETF | BGI | FLOCK | R | VUL | - | 0.0010 | 0.0000 |
| Rufous Woodpecker | *Celeus brachyurus* | FS | BGI | FLOCK | R | VUL | - | 0.0003 | 0.0000 |
| Grey-headed Woodpecker | *Picus canus* | ETF | BGI | FLOCK | R | VUL | - | 0.0007 | 0.0000 |
| Bay Woodpecker | *Blythipicus pyrrhotis* | ETF | BGI | FLOCK | R | VUL | - | 0.0010 | 0.0000 |
| Blue-winged Pitta | *Pitta moluccensis* | ETF | TI | NON | R | VUL | - | 0.0003 | 0.0000 |
| Ashy Minivet | *Pericrocotus divaricatus* | ETF | AFGI | MONO | P | VUL | - | 0.0000 | 0.0066 |
| Scarlet Minivet | *Pericrocotus flammeus* | ETF | AFGI | FLOCK | R | VUL | S12 | 0.0144 | 0.0159 |
| Grey-chinned Minivet | *Pericrocotus solaris* | ETF | AFGI | FLOCK | R | VUL | S33 | 0.0459 | 0.1084 |
| Long-tailed Shrike | *Lanius schach* | OS | SI | NON | R | RES | S49 | 0.0027 | 0.0013 |
| Red-billed Blue Magpie | *Urocissa erythrorhyncha* | ETF | AFGIF | NON | R | VUL | S28 | 0.0068 | 0.0141 |
| Gray Treepie | *Dendrocitta formosae* | FS | AFGIF | MONO | R | VUL | S36 | 0.0130 | 0.0044 |
| Great Tit | *Parus major* | G | AFGI | FLOCK | R | MODER | S14 | 0.0051 | 0.0040 |
| Black-throated Tit | *Aegithalos concinnus* | FS | AFGI | FLOCK | R | VUL | S23 | 0.0301 | 0.0414 |
| Hill Prinia | *Prinia atrogularis* | ES | AFGI | NON | R | VUL | S19 | 0.0031 | 0.0022 |
| Yellow-bellied Prinia | *Prinia flaviventris* | OS | AFGI | NON | R | RES | - | 0.0010 | 0.0000 |
| Plain Prinia | *Prinia inornata* | OS | AFGI | NON | R | RES | - | 0.0000 | 0.0026 |
| Common Tailorbird | *Orthotomus sutorius* | OS | AFGI | NON | R | RES | - | 0.0000 | 0.0009 |
| Collared Finchbill | *Spizixos semitorques* | ETF | AFGIF | MONO | R | VUL | S41 | 0.0072 | 0.0088 |
| Light-vented Bulbul* | *Pycnonotus sinensis* | OS | AFGIF | MONO | R | RES | S6 | 0.0154 | 0.0379 |
| Red-whiskered Bulbul* | *Pycnonotus jocosus* | OS | AFGIF | MONO | R | RES | S22 | 0.0038 | 0.0101 |
| Mountain Bulbul | *Ixos mcclellandii* | FS | AFGIF | FLOCK | R | VUL | S40 | 0.0151 | 0.0044 |
| Black Bulbul | *Hypsipetes leucocephalus* | FS | AFGIF | MONO | R | VUL | S18 | 0.2222 | 0.0727 |
| Chestnut Bulbul | *Hemixos castanonotus* | FS | AFGIF | FLOCK | R | VUL | S38 | 0.0294 | 0.0370 |
| Brownish-flanked Bush Warbler | *Cettia fortipes* | ETF | AFGI | NON | R | VUL | - | 0.0003 | 0.0000 |
| Dusky Warbler | *Phylloscopus fuscatus* | G | AFGI | NON | W | VUL | S17 | 0.0010 | 0.0009 |
| Hume’s Leaf Warbler | *Phylloscopus humei* | FS | AFGI | FLOCK | W | VUL | S29 | 0.0031 | 0.0009 |
| Pallas's Leaf Warbler | *Phylloscopus proregulus* | FS | AFGI | FLOCK | W | VUL | S30 | 0.0092 | 0.0093 |
| Chestnut-crowned Warbler | *Seicercus castaniceps* | FS | AFGI | FLOCK | R | VUL | - | 0.0003 | 0.0000 |
| Masked Laughingthrush | *Garrulax perspicillatus* | OS | TI | MONO | R | VUL | S20 | 0.0027 | 0.0088 |
| Greater Necklaced Laughingthrush | *Garrulax pectoralis* | FS | TI | MONO | R | VUL | S21 | 0.0342 | 0.0674 |
| Lesser Necklaced Lauhgingthrush | *Garrulax monileger* | FS | TI | MONO | R | VUL | S46 | 0.0058 | 0.0110 |
| Hwamei | *Garrulax canorus* | FS | TI | FLOCK | R | VUL | - | 0.0000 | 0.0004 |
| Red-billed Leiothrix | *Leiothrix lutea* | FS | AFGI | FLOCK | R | VUL | - | 0.0075 | 0.0000 |
| Rufous-capped Babbler | *Stachyris ruficeps* | FS | AFGI | FLOCK | R | VUL | S24 | 0.0048 | 0.0040 |
| Huet’s Fulvetta | *Alcippe huet* | FS | AFGI | FLOCK | R | VUL | S35 | 0.3769 | 0.3861 |
| Striated Yuhina | *Yuhina castaniceps* | FS | AFGI | FLOCK | R | VUL | S39 | 0.0445 | 0.0661 |
| Rusty-cheeked Scimitar Babbler | *Pomatorhinus erythrogenys* | FS | AFGI | FLOCK | R | VUL | S47 | 0.0031 | 0.0018 |
| Streak-breasted Scimitar Babbler | *Pomatorhinus ruficollis* | FS | AFGI | FLOCK | R | VUL | S50 | 0.0045 | 0.0009 |
| White-bellied Yuhina | *Erpornis zantholeuca* | FS | AFGI | FLOCK | R | VUL | S3 | 0.0024 | 0.0004 |
| Grey-headed Parrotbill | *Paradoxornis gularis* | FS | AFGIF | MONO | R | VUL | - | 0.0003 | 0.0000 |
| Japanese Whit-eye | *Zosterops japonicus* | OS | AFGI | MONO | R | RES | S1 | 0.0017 | 0.0035 |
| Siberian Rubythroat | *Luscinia calliope* | ES | AFGI | NON | P | VUL | - | 0.0003 | 0.0000 |
| White-crowned Forktail | *Enicurus leschenaulti* | FS | SI | NON | R | VUL | S2 | 0.0021 | 0.0022 |
| Slaty-backed Forktail | *Enicurus schistaceus* | FS | SI | NON | R | VUL | S32 | 0.0055 | 0.0071 |
| Daurian Redstart | *Phoenicurus auroreus* | G | AFGI | NON | W | VUL | S9 | 0.0010 | 0.0031 |
| Plumbeous Water Redstart | *Rhyacornis fuliginosa* | FS | SI | NON | R | VUL | S26 | 0.0038 | 0.0018 |
| Orange-flanked Bush Robin* | *Luscinia cyanura* | ETF | AFGI | NON | W | VUL | S27 | 0.0079 | 0.0031 |
| Grey-backed Thrush | *Turdus hortulorum* | ETF | TIF | NON | W | VUL | S31 | 0.0034 | 0.0009 |
| Oriental Magpie Robin | *Copsychus saularis* | OS | AFGI | NON | R | RES | S43 | 0.0003 | 0.0004 |
| Blue Whistling Thrush | *Myophonus caeruleus* | ETF | TIF | NON | S | VUL | S48 | 0.0010 | 0.0004 |
| Asian Brown Flycatcher | *Muscicapa dauurica* | ETF | SI | NON | W | VUL | - | 0.0007 | 0.0000 |
| Blue-and white Flycatcher | *Cyanoptila cyanomelana* | FS | SI | NON | P | VUL | - | 0.0000 | 0.0013 |
| Fujian Niltava | *Niltava davidi* | FS | SI | NON | W | VUL | - | 0.0003 | 0.0000 |
| Brown Dipper | *Cinclus pallasii* | FS | MIP | NON | R | VUL | - | 0.0024 | 0.0000 |
| Orange-bellied Leafbird | *Chloropsis hardwickei* | ETF | NIF | FLOCK | R | VUL | S11 | 0.0021 | 0.0022 |
| Fire-breasted Flowerpecker | *Dicaeum ignipectum* | ETF | NIF | FLOCK | R | VUL | - | 0.0000 | 0.0018 |
| Fork-tailed Sunbird | *Aethopyga christinae* | ETF | NIF | FLOCK | R | VUL | S10 | 0.0007 | 0.0026 |
| White-rumped Munia | *Lonchura striata* | OS | TF | MONO | R | RES | S7 | 0.0031 | 0.0018 |
| Scaly-breasted Munia | *Lonchura punctulata* | OS | TF | MONO | R | RES | - | 0.0003 | 0.0000 |
| White Wagtail | *Motacilla alba* | OS | SI | NON | R | RES | S4 | 0.0045 | 0.0062 |
| Grey Wagtail | *Motacilla cinerea* | OS | SI | NON | W | VUL | S34 | 0.0007 | 0.0018 |
| Olive-backed Pipit | *Anthus hodgsoni* | ES | TI | MONO | W | VUL | S45 | 0.0106 | 0.0115 |
| Yellow-billed Grosbeak | *Eophona migratoria* | OS | TF | MONO | W | RES | - | 0.0086 | 0.0000 |
| Slaty Bunting | *Latoucheornis siemsseni* | ES | TF | NON | W | VUL | - | 0.0000 | 0.0004 |
| Tristram's Bunting | *Emberiza tristrami* | ES | TF | NON | W | VUL | S5 | 0.0007 | 0.0013 |
| Black-faced Bunting | *Emberiza spodocephala* | OS | TF | MONO | W | RES | S37 | 0.0007 | 0.0031 |
| Little Bunting | *Emberiza pusilla* | OS | TF | MONO | W | RES | - | 0.0000 | 0.0026 |
| Yellow-browed Bunting | *Emberiza chrysophrys* | OS | TF | NON | W | RES | - | 0.0024 | 0.0000 |

**^a^** Habitat preference: “edge species (ES)”; “edge-tolerant forest species (ETF)”; forest specialist (FS)”; “generalist (G)”; “open-habitat species (OS)”.

**^b^** Dietary guilds: “arborous foliage glean insectivore (AFGI)”; “arborous foliage glean insectivore-frugivore (AFGIF)”; “arboreal frugivore–predator (AFP)”; “bark-gleaning insectivore (BGI)”; “miscellaneous insectivore-piscivore (MIP)”; “nectarivores-insectivore-frugivore (NIF)”; “sallying insectivore (SI)”; “terrestrial frugivore (TF)”; “terrestrial insectivore (TI)”; “terrestrial insectivore-frugivore (TIF)”.

**^c^** Flocking guild: “mixed-species flock participants (FLOCK)”; “Non-flocking species (NON)” and “monospecific flock participants (MONO)”.

^d^ Migratory status: “passage migrant (P)”; “permanent resident (R)”; “summer visitor (S)” and “winter visitor (W)”.

**^e^** Human tolerance: “moderate susceptibility (MODER)”; “vulnerable to human disturbance (VUL)” and “resistant to human disturbance (RES)”.

^f^ Code represents the abbreviation for species used in CCA ordination (Fig. 3).

* indicating a significant individual change between pre- and post-storm at *P* < 0.05.

**Table S2. Perching height of fifty bird species during pre- and post-storm and statistical tests.** The T values are derived from Paired T-Test. Level of significance: * *P* < 0.05; ** *P* < 0.01. ^a^ Sampling size: the number of censuses in which the given species were seen.

| **Species name** | **Pre-storm** | | **Post-storm** | | **T Test** |
| --- | --- | --- | --- | --- | --- |
|  | **Perching height (m)** | **Sampling size^a^** | **Perching height (m)** | **Sampling size^a^** |  |
| Common Coucal | 5.00±5.00 | 2 | 0.00 | 1 | *T* =0.577, *df*=1, *P*=0.667 |
| Red-headed Trogon | 4.75±0.93 | 6 | 1.83±0.40 | 6 | *T*=2.883, *df*=10, *P*=0.016* |
| Dollarbird | 50.50±13.89 | 6 | 5.00 | 1 | *T*=1.238, *df*=5, *P=*0.271 |
| Blyth's Kingfisher | 1.17±0.17 | 3 | 2.00 | 1 | *T*=-2.500, *df*=2, *P=*0.130 |
| Common Kingfisher | 1.67±0.67 | 3 | 3.00 | 1 | *T*=-1.000, *df*=2, *P=*0.423 |
| Crested Kingfisher | 4.50±0.65 | 4 | 4.00 | 1 | *T*=0.346, *df*=3, *P=*0.752 |
| Great Barbet | 12.50±2.50 | 2 | 8.00 | 1 | *T*=1.039, *df*=1, *P=*0.488 |
| Scarlet Minivet | 17.00±2.72 | 5 | 9.00±1.29 | 4 | *T*=2.434, *df*=7, *P=*0.045* |
| Grey-chinned Minivet | 15.63±1.84 | 8 | 11.50±1.40 | 8 | *T*=1.783, *df*=14, *P=0*.096 |
| Long-tailed Shrike | 5.44±1.69 | 8 | 2.17±0.93 | 3 | *T*=1.125, *df*=9, *P=*0.290 |
| Red-billed Blue Magpie | 1.33±1.15 | 6 | 2.78±1.09 | 9 | *T*=-0.884, *df*=13, *P=*0.393 |
| Gray Treepie | 18.60±0.30 | 15 | 21.33±10.41 | 3 | *T*=-0.232, *df*=16, *P=*0.820 |
| Great Tit | 7.38±2.07 | 8 | 7.80±1.80 | 5 | *T*=-0.254, *df=*11, *P=*0.804 |
| Black-throated Tit | 10.86±2.08 | 7 | 8.40±2.93 | 5 | *T*=0.707, *df=*10, *P=*0.496 |
| Hill Prinia | 0.64±0.09 | 7 | 0.83±0.17 | 3 | *T*=-1.079, *df*=8, *P=*0.312 |
| Collared Finchbill | 11.18±3.36 | 11 | 9.41±4.16 | 11 | *T*=0.331, *df*=20, *P=*0.744 |
| Light-vented Bulbul | 8.90±4.05 | 7 | 5.25±1.50 | 8 | *T*=0.890, *df*=13, *P=*0.390 |
| Red-whiskered Bulbul | 5.00±2.46 | 6 | 3.68±0.93 | 11 | *T*=0.603, *df*=15, *P=*0.555 |
| Mountain Bulbul | 12.46±3.93 | 12 | 9.29±1.67 | 7 | *T*=0.592, *df=*17, *P=*0.562 |
| Black Bulbul | 23.29±1.85 | 41 | 13.29±1.43 | 14 | *T*=3.040, *df=*53, *P=*0.004** |
| Chestnut Bulbul | 11.16±1.79 | 16 | 10.85±2.11 | 13 | *T*=0.113, *df=*27, *P=*0.911 |
| Dusky Warbler | 0.50 | 3 | 0.50 | 2 | NA |
| Hume’s Leaf Warbler | 7.50±1.02 | 6 | 8.00 | 1 | *T=*-0.184, *df*=5, *P*=0.861 |
| Pallas's Leaf Warbler | 7.55±1.77 | 11 | 4.00±0.68 | 6 | *T=*1.433, *df*=15, *P*=0.173 |
| Masked Laughingthrush | 4.00±4.00 | 2 | 3.00 | 1 | *T=*0.144, *df=*1, *P*=0.909 |
| Greater Necklaced Laughingthrush | 3.43±1.29 | 7 | 2.88±0.72 | 8 | *T=*0.388, *df*=13, *P*=0.704 |
| Lesser Necklaced Lauhgingthrush | 7.50±7.50 | 2 | 10.00 | 1 | *T=*-0.192, *df*=1, *P*=0.879 |
| Rufous-capped Babbler | 4.15±1.10 | 10 | 1.75±0.48 | 4 | *T=*1.329, *df=*12, *P*=0.208 |
| Huet’s Fulvetta | 3.10±0.30 | 66 | 2.20±0.20 | 49 | *T=*2.268, *df*=113, *P*=0.025* |
| Striated Yuhina | 17.33±11.57 | 3 | 6.00±2.00 | 3 | *T*=0.966, *df*=4, *P=*0.389 |
| Rusty-cheeked Scimitar Babbler | 0.56±0.20 | 5 | 0.75±0.25 | 2 | *T*=-0.536, *df=*5, *P=*0.615 |
| Streak-breasted Scimitar Babbler | 2.64±1.32 | 7 | 1.00 | 1 | *T*=0.441, *df*=6, *P=*0.675 |
| White-bellied Yuhina | 8.50±2.66 | 6 | 1.00 | 1 | *T*=1.068, *df=*5, *P=*0.335 |
| Japanese Whit-eye | 4.00 | 1 | 12.00 | 2 | NA |
| White-crowned Forktail | 0.25±0.25 | 4 | 0.20±0.20 | 5 | *T*=0.158, *df=*7, *P=*0.879 |
| Slaty-backed Forktail | 0.03±0.03 | 12 | 0.10±0.10 | 10 | *T*=-0.790, *df=*20, *P=*0.439 |
| Daurian Redstart | 3.50±1.32 | 3 | 1.17±0.21 | 6 | *T*=2.538, *df=*7, *P=*0.039* |
| Plumbeous Water Redstart | 0.74±0.48 | 10 | 0.25±0.14 | 4 | *T*=0.629, *df=*12, *P=*0.541 |
| Orange-flanked Bush Robin | 1.35±0.31 | 22 | 1.14±0.24 | 7 | *T=*0.366, *df=*27, *P=*0.717 |
| Grey-backed Thrush | 8.50±3.62 | 3 | 3.50±2.50 | 2 | *T=*0.994, *df=*3, *P=*0.393 |
| Oriental Magpie Robin | 8.00 | 1 | 0.50 | 1 | NA |
| Blue Whistling Thrush | 1.67±1.67 | 3 | 0.00 | 1 | *T=*0.500, *df=*2, *P=*0.667 |
| Orange-bellied Leafbird | 7.17±4.23 | 3 | 10.50±2.10 | 4 | *T=*-0.771, *df*=5, *P=*0.475 |
| Fork-tailed Sunbird | 0.50 | 1 | 2.80±1.32 | 5 | *T=*-0.712, *df*=4, *P=*0.516 |
| White-rumped Munia | 0.60±0.40 | 2 | 1.00 | 1 | *T=*-0.577, *df=*1, *P=*0.667 |
| White Wagtail | 1.67±1.67 | 9 | 0.70±0.47 | 10 | *T=*0.585, *df=*17, *P=*0.566 |
| Grey Wagtail | 0.00±0.00 | 2 | 0.13±0.13 | 4 | *T=*-0.667, *df=*4, *P=*0.541 |
| Olive-backed Pipit | 6.55±2.41 | 10 | 2.86±0.86 | 7 | *T=*1.233, *df=*15, *P=*0.237 |
| Tristram's Bunting | 1.00±0.00 | 2 | 0.75±0.25 | 2 | *T=*1.000, *df=*2, *P=*0.423 |
| Black-faced Bunting | 1.50 | 1 | 2.67±1.67 | 3 | *T=*-0.350, *df=*2, *P=*0.760 |
